# Rolling Bearing Fault Diagnosis Based on Support Vector Machine Optimized by Improved Grey Wolf Algorithm

**DOI:** 10.3390/s23146645

**Published:** 2023-07-24

**Authors:** Weijie Shen, Maohua Xiao, Zhenyu Wang, Xinmin Song

**Affiliations:** 1 Zhejiang Technical Institute of Economics, Hangzhou 310018, China; 240056@zjtie.edu.cn; 2 College of Engineering, Nanjing Agricultural University, Nanjing 210031, China; 2021812053@stu.njau.edu.cn (Z.W.); 9203012015@stu.njau.edu.cn (X.S.)

**Keywords:** rolling bearing, fault diagnosis, IGWO algorithm, SVM algorithm

## Abstract

This study targets the low accuracy and efficiency of the support vector machine (SVM) algorithm in rolling bearing fault diagnosis. An improved grey wolf optimizer (IGWO) algorithm was proposed based on deep learning and a swarm intelligence optimization algorithm to optimize the structural parameters of SVM and improve the rolling bearing fault diagnosis. A nonlinear contraction factor update strategy was also proposed. The variable coefficient changes with the shrinkage factor *α*. Thus, the search ability was balanced at different early and late stages by controlling the dynamic changes of the variable coefficient. In the early stages of optimization, its speed is low to avoid falling into local optimization. In the later stages of optimization, the speed is higher, and finding the optimal solution is easier, balancing the two different global and local optimization capabilities to complete efficient convergence. The dynamic weight update strategy was adopted to perform position updates based on adaptive dynamic weights. First, the dataset of Case Western Reserve University was used for simulation, and the results showed that the diagnosis accuracy of IGWO-SVM was 98.75%. Then, the IGWO-SVM model was trained and tested using data obtained from the full-life-cycle test platform of mechanical transmission bearings independently researched and developed by Nanjing Agricultural University. The fault diagnosis accuracy and convergence value of the adaptation curve were compared with those of PSO-SVM (particle swarm optimization) and GWO-SVM diagnosis models. Results showed that the IGWO-SVM model had the highest rolling bearing fault diagnosis accuracy and the best diagnosis convergence.

## 1. Introduction

Rolling bearings are widely used and critical in mechanical engineering. Rolling bearings convert the sliding friction between shafts into rolling friction through rolling rotors, reducing the energy loss and lowering the operating speed of rotating parts. They are used in rotating parts of different mechanical equipment. However, rolling bearings work under extremely complex and harsh environments, high speed, high temperature, and heavy load. Thus, they are prone to fatigue damage, significantly hindering the operation of mechanical systems, and may lead to huge economic losses and casualties. Thus, fault diagnosis is of great significance for rolling bearings. Predicting and diagnosing rolling bearing mechanical faults can prevent faults and damage to mechanical equipment and provide important technical support for maintenance [1,2,3,4].

Rolling bearing fault diagnosis includes multiple processes of vibration signal data acquisition, data preprocessing, feature extraction, and fault identification [5,6,7,8]. During research on fault feature extraction, rolling bearing fault vibration signal features are commonly extracted based on signal time-domain features [9]. Signal time-domain features are widely used because of their simple and fast calculation, higher time precision, and accuracy. Features of the signal time domain include extracting dimensional and dimensionless feature parameters. Dimensional feature parameters have strong sensitivity but poor stability, whereas dimensionless feature parameters have strong stability but poor sensitivity [10,11]. Mechanical fault signal features of rolling bearings can be extracted by combining dimensional feature parameter extraction with dimensionless feature parameter extraction. During research on fault identification, with the continuous optimization and development of deep learning and intelligent optimization algorithms in recent years, swarm intelligence optimization algorithms have been effectively applied to rolling bearing fault diagnosis, which has become a hotspot in this field. Artificial neural networks [12], backpropagation (BP) neural networks [13], and support vector machines (SVMs) [14] are common machine learning algorithms. An artificial neural network is prone to overfitting, a BP neural network tends to fall into local optimality [15,16], and an SVM effectively solves the above problems. With their sound learning performance, SVMs are widely used in fault diagnosis and identification [17,18].

However, in fault identification, the structural parameter selection of the support vector machine has a great influence on the calculation result. The selection of structural parameters based on artificial experience methods has certain randomness and tendency, and it is difficult to select appropriate structural parameters. Therefore, swarm intelligence optimization algorithms such as the genetic algorithm, particle swarm optimization (PSO) algorithm, and grey wolf optimization (GWO) algorithm are used to solve this problem. Swarm algorithms feature good optimization performance and easy implementation and can be effectively used to optimize the structural parameters of SVMs [19]. Proposed by García et al. [20], the PSO-SVM model differs from traditional SVM models; it improves the accuracy of the prediction of the remaining service life of aircraft engines. Dong et al. [21] proposed the GWO-SVM model to improve the efficiency of rolling bearing fault diagnosis. However, the experimental calculation results show that the PSO algorithm is prone to falling into local optimality [22], the GWO algorithm has low optimization accuracy, and simple swarm algorithms cannot meet operational standards. Thus, the optimization algorithm can be considered to adjust the optimization accuracy. Therefore, the GWO algorithm must be improved.

An improved GWO (IGWO) model is proposed in this study to enhance the GWO algorithm and improve the classification effect of an SVM. The GWO algorithm is mainly used to search for optimization by imitating the hunting behavior of grey wolves and has been applied in many fields [23]. Song Yusheng et al. used IGWO to optimize an SVM. They aimed at the problems of low efficiency and easily falling into a local optimum in GWO optimization and improved the traditional GWO model through nonlinear control and random weight position updating [24]. The test results showed that the model effectively improved the fault diagnosis efficiency and recognition rate. Yu Han proposed an improved GWO algorithm to solve the economic dispatching of power systems [25]. He introduced the inertia characteristic constant and Gaussian mutation operator to search for the optimal value and compared it with the PSO algorithm. The results show that this method has the advantages of fast convergence, high accuracy, and ease of jumping out of the optimal local solution. Therefore, improving the GWO algorithm can improve final diagnosis efficiency and accuracy.

The above research proposed an SVM-based rolling bearing mechanical fault diagnosis method based on the optimized GWO algorithm. First, targeting the low accuracy and efficiency of the SVM algorithm in rolling bearing fault diagnosis, an improved GWO algorithm based on deep learning and swarm intelligence optimization algorithm was proposed, and the structural parameters of the SVM were optimized based on the IGWO algorithm to improve the rolling bearing fault diagnosis effect. Second, four dimensional feature parameters and four dimensionless feature parameters of the vibration signals were extracted as those of the vibration signals to extract the vibration signal features accurately. Finally, the IGWO-SVM model simulation training and testing were carried out based on the test data of the full-life-cycle test platform for mechanical transmission system bearings at Nanjing Agricultural University, and the fault diagnosis accuracy and convergence value of adaptation curve were compared with those of PSO-SVM and GWO-SVM diagnosis models. The results showed that the IGWO-SVM model had the maximum rolling bearing fault diagnosis accuracy, the best diagnosis convergence, and the best algorithm performance.

## 2. Theoretical Principle

### 2.1. Grey Wolf Optimizer

In 2014, Mirjalili et al. proposed an intelligent population algorithm, the GWO algorithm [26]. Its main advantages are strong convergence, few parameters, and easy implementation, and it has been widely used in parameter optimization, power scheduling, image processing, and other fields. GWO can solve various optimization problems and search for the global optimal solution more effectively [27].

**Figure 1:**
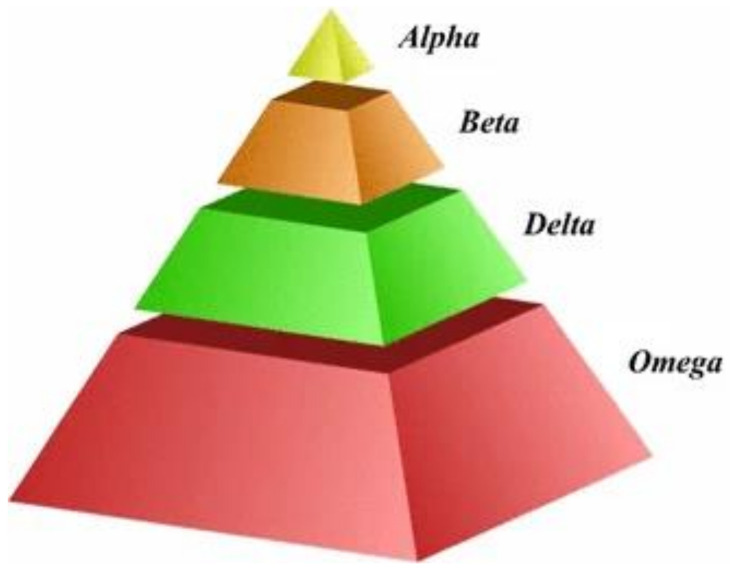
Social hierarchy of grey wolves (figure provided by Grey wolf optimizer: a review of recent variants and applications).

GWO is a kind of swarm intelligence technique. The GWO algorithm was inspired by the grey wolf’s social intelligence in leadership and hunting. The power and domination of grey wolf packs follow the same social hierarchy [28]. Figure 1 shows that the GWO algorithm divides the wolf pack into α, β, δ, and ω, among which the grey wolves α, β, and δ have levels and division of labor in the wolf pack and mainly lead and direct the whole process of predation by the wolf pack. The grey wolf ω is at the mercy of its superiors during hunting. The GWO algorithm is divided into three processes: encircling, hunting, and attacking [29,30].

1. Encircling

Encircling generates a group of random initial populations. The fitness function of each individual is then calculated, the position of the leader wolf is determined, and the distance between each individual and the leader wolf is calculated. Each individual moves according to the position between the individual and the leader wolf. During encircling, each grey wolf moves according to the following formulas:

(1) $$D=\left|CX_{p\left(t\right)}-X_{t}\right|,$$

(2) $$X_{t+1}=X_{p\left(t\right)}-AD,$$

where *D* represents the Euclidean distance between the individual grey wolf and the prey, *X_p(t)_* represents the position of the prey, *X_t_* represents the individual position of the grey wolf before the process of encircling the prey begins, and *X_t+_*_1_ represents the individual position of the grey wolf after encircling the prey.

The computation formulas for the variable coefficients *A* and *C* which play a role in the computation of certain parameters and variables within the algorithm are as follows:

(3) $$A=2ar_{1}-a,$$

(4) $$C=2r_{2},$$

where *a* represents the contraction factor, decreasing linearly from 2 to 0, and *r_1_* and *r_2_* represent two random numbers in the interval [0, 1].

2. Hunting

**Figure 2:**
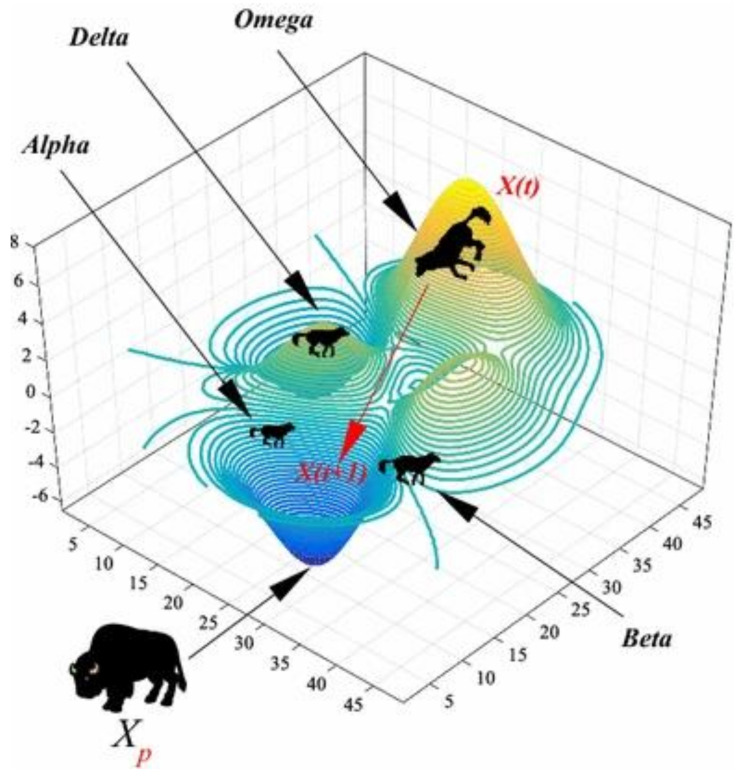
How alpha, beta, delta, and omega are defined in GWO (figure provided by GWO: a review of recent variants and applications).

The fitness function of the individual grey wolf is calculated after the stage of encircling the prey. The prey is hunted after movement with the grey wolf α, β, and δ as the leaders. The movement continues according to the distance between each individual and the leader wolf. Each grey wolf in the hunting process moves according to the following formulas:

(5) $$D_{q}=\left|C_{l}X_{j}-X_{f\left(t\right)}\right|,$$

(6) $$X_{l}=X_{q}-A_{l}D_{q},$$

(7) $$X_{f\left(t+1\right)}=\frac{\sum X_{o}}{3},$$

where q selects α, β, and δ, and *l* selects 1, 2, and 3. Formula (5) calculates the difference (absolute value) between the product of *C_l_* and *X_j_* and the value of *X_f(t)_*. This calculation is used to quantify the discrepancy or distance between the two values, which likely plays a role in the optimization process or analysis being conducted. Here, *D_q_* represents the Euclidean distance between q wolf and the individual grey wolf. *X_j_* represents the position of the gray wolf during the chase. It represents a specific attribute or feature of the data being analyzed, which could refer to a particular dimension or variable within the dataset that is relevant to the calculation of *D_q_*. *X_l_* represents the distance that an individual grey wolf moves to q wolf. *X_f(t)_* represents the individual location of the grey wolf before hunting begins. *X_f(t+_*_1)_ represents the individual position of the grey wolf after hunting. The variable coefficients *A_l_* and *C_l_* are determined in the same way as Formulas (3) and (4) [28]. Figure 2 shows the image description of the algorithm in related literature.

3. Attacking

After the wolf pack decides to attack the prey, according to Formula (3), when a linearly decreases from 2 to 0, the value range of *A* is [−a, a]. When the absolute value of *A* is less than or equal to 1, the grey wolves will attack the prey. When the absolute value of *A* is greater than 1, the grey wolves will leave the wolf pack to search for the next prey, expanding the search range of the entire wolf pack. 

**Figure 3:**
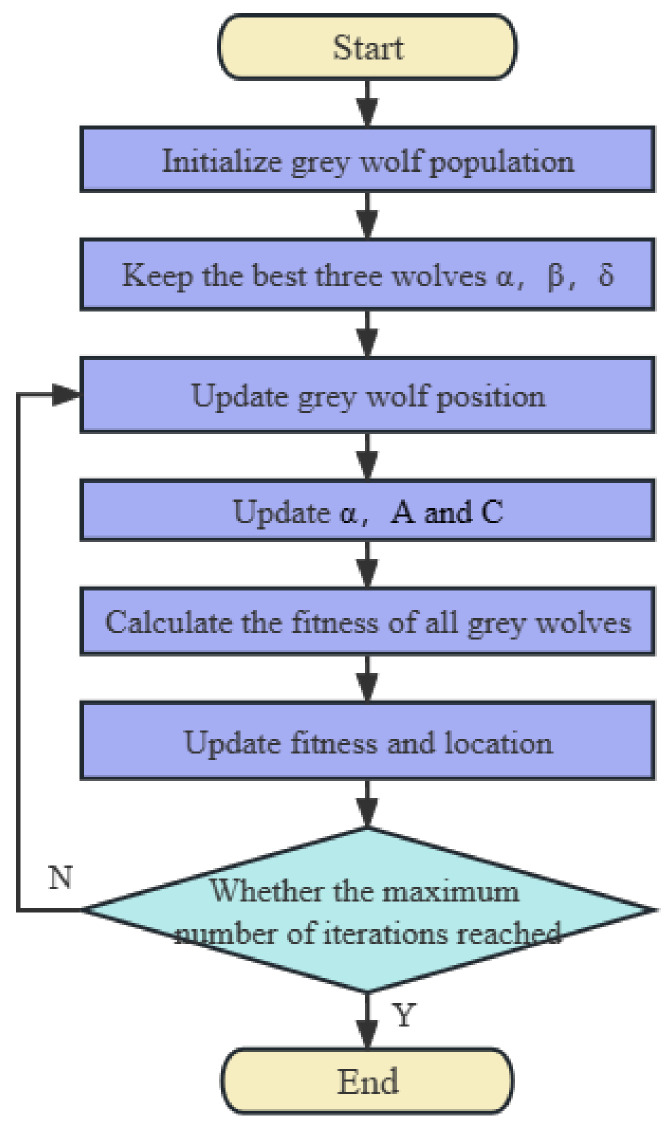
Flow chart of the GWO algorithm.

The specific flow of the GWO algorithm is shown in Figure 3.

### 2.2. Optimization Process

#### 2.2.1. Control Parameter Optimization

Given that the initial population of the standard GWO algorithm is generated by random initialization, the initial population is randomly distributed in the solution space, and the uniformity of the population distribution is poor, so the initial population cannot cover the entire solution space, which directly affects the search efficiency and optimization accuracy of the GWO algorithm. 

In the standard GWO algorithm, the shrinkage factor a decreases linearly from 2 to 0. Therefore, at the initial optimization stage of the GWO algorithm, the shrinkage factor a is relatively large, and this algorithm executes the global search. In the later stage of algorithm optimization, the shrinkage factor a decreases as the number of iterations increases, and this algorithm executes the local search. 

In the GWO algorithm, the control parameter a decreases linearly, and the optimization process of the GWO algorithm is nonlinear. A linear update strategy cannot maximize the global search ability and local development ability of the GWO algorithm. On the contrary, it may fall into local optimality. 

A nonlinear shrinkage factor update strategy is proposed to improve the quality of the initial population distribution of the standard GWO algorithm and increase the uniformity and diversity of the initial population distribution. Formula (3) shows that the value of A changes with the value of a. Thus, the dynamic change in a should be controlled to balance the search ability of different stages in the early and late stages. The calculation formula of a value is shown in Formula (8). In the early stage of optimization, the speed is low to avoid falling into local optimization. In the later stage of optimization, the speed is higher, and the optimal solution is easier to find, effectively balancing the global and local optimization capabilities and thus achieving efficient convergence.

(8) $$a\left(t\right)=2.5exp(0.2(-\log\frac{8t}{T}),$$

where *T* and *t* represent the maximum and current numbers of iterations. The specific coefficients (2.5, 0.2, and 8) have been chosen based on empirical knowledge or prior research. These values may have been determined through experimentation or optimization to achieve desirable algorithm performance.

#### 2.2.2. Position Update Optimization

Each update position of the GWO algorithm is determined by assigning the same weight to the positions of α, β, and δ wolves, but their features and decision-making abilities are different, resulting in a slow convergence speed of the algorithm and the inability to obtain a globally optimal solution. 

This study applies the dynamic weight update strategy to update the position based on the adaptive dynamic weight. The formulas are as follows:

(9) $$w_{i}=\left|\frac{x_{i}}{x_{1}+x_{2}+x_{3}}\right|i\in \left\{1,2,3\right\},$$

(10) $$w_{j}=\left|\frac{f_{j}}{f_{\alpha }+f_{\beta }+f_{\delta }}\right|j\in \left\{\alpha ,\beta ,\delta \right\},$$

(11) $$x_{t+1}=(\frac{2}{t_{max}}+1)\times \frac{w_{\alpha }w_{1}x_{1}+w_{\beta }w_{2}x_{2}+w_{\delta }w_{3}x_{3}}{3},$$

where *x_i_* is the current distance that w wolf needs to move toward α, β, and δ wolves; *t_max_* represents the maximum number of iterations in the optimization process. It denotes the predefined or chosen limit for the iterations before termination. The coefficient $\frac{2}{t_{max}}+1$ controls the weight of the term $\frac{w_{\alpha }w_{1}x_{1}+w_{\beta }w_{2}x_{2}+w_{\delta }w_{3}x_{3}}{3}$ in the formula, and it changes according to the current iteration (*t*) and the maximum number of iterations (*t_max_*); *w_j_* is the position weight of *w* wolf for these three wolves; and *f_j_* is the fitness value of α, β, and δ wolves. Each updated position of the individual grey wolf is shown in Formula (11). 

### 2.3. IGWO-SVM Fault Diagnosis Model

The GWO algorithm has good global optimization and has the defect of difficult convergence. Based on the features of the above algorithm, its structure is improved; thus, the IGWO algorithm is proposed accordingly. 

The proposed approach integrates the improved grey wolf optimizer (IGWO) algorithm with support vector machine (SVM) training to enhance the fault diagnosis performance. The interaction between IGWO and SVM training is a two-step process aimed at optimizing the structural parameters of SVM for improved classification accuracy.

In the first step, the IGWO algorithm is employed to optimize the parameters of SVM, such as the penalty factor (c) and kernel function parameter (g). IGWO, inspired by the hunting behavior of grey wolves, exhibits excellent exploration and exploitation abilities. It employs a population-based search strategy to iteratively update the parameter values based on fitness evaluation. This optimization process seeks to find the optimal combination of c and g that maximizes the classification accuracy of the SVM model.

Once the optimal parameter values are obtained from the IGWO optimization, the second step involves training the SVM model using the training dataset. The SVM classifier utilizes the optimized parameters to build a decision boundary that effectively separates different fault classes. The training process involves mapping the input data to a higher-dimensional feature space and determining the hyperplane that maximally separates the classes. The training algorithm adjusts the SVM model to minimize classification errors and maximize the margin between support vectors.

The integration of IGWO and SVM training enables the identification and utilization of optimal parameter values for improved fault diagnosis. The IGWO algorithm efficiently explores the parameter space, while SVM training ensures the model learns the discriminative patterns present in the data. By optimizing the SVM parameters through IGWO and training the SVM model with the optimized parameters, the proposed approach achieves enhanced classification accuracy in rolling bearing fault diagnosis.

A comparison of the algorithm flow between the grey wolf optimizer (GWO) and the improved grey wolf optimizer (IGWO) is presented as follows:

(1) GWO algorithm flow:
  - Initialization: Randomly initialize the positions and fitness of a group of grey wolf individuals.
  - Update Alpha, Beta, and Delta Wolves: Update the positions of Alpha, Beta, and Delta wolves based on their fitness values.
  - Update Other Wolves’ Positions: Update the positions of the remaining wolves based on the positions of Alpha, Beta, and Delta wolves.
  - Boundary Handling: Perform boundary handling on the updated wolf positions to ensure they fall within the defined problem range.
  - Update Fitness: Calculate the fitness values of the updated wolf individuals.
  - Termination
(2) IGWO algorithm flow (additional Steps):
  - Nonlinear Contraction Factor Update: Introduce a nonlinear contraction factor update strategy to balance the search ability at different stages of optimization.
  - Dynamic Weight Update: Incorporate a dynamic weight update strategy for position updates based on adaptive dynamic weights.

These additional steps in the IGWO algorithm, the nonlinear contraction factor update and the dynamic weight update, are the key differences in the algorithm flow compared to the GWO algorithm.

**Figure 4:**
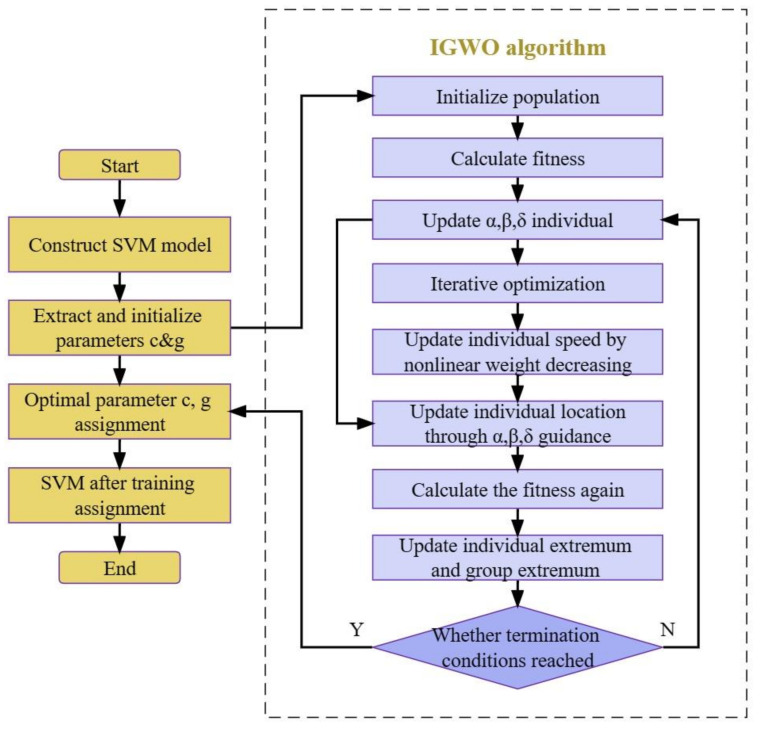
IGWO-SVM fault diagnosis model.

An SVM is an effective machine learning model. Its core function is to construct a hyperplane through data based on samples, classify sample data, and maintain the maximum distance between sample points and hyperplanes [31]. It features easy implementation and good robustness. According to the research of scholars, in the process of SVM training, the results show that the SVM penalty factor c and the RBF kernel function parameter g have a great impact on the performance of the SVM; penalty factor c represents the fault tolerance degree in the classification process and represents the relationship between SVM classification accuracy and complexity [32,33]. The larger the value of c is, the higher the final classification accuracy will be, but the problem of overfitting will also occur. When the value of c is smaller, the classification accuracy will decrease, and the underfitting problem will easily occur. The kernel function g represents the mapping relationship between raw data and higher-dimensional data. The higher the value of g, the higher the classification accuracy, but the hyperplane will become complicated. The smaller the value of g, the higher the probability of misjudgment in classification accuracy. Thus, the values of the two must be balanced, which is conducive to the generalization and promotion of the model. Therefore, this study proposes an IGWO-SVM fault diagnosis model based on SVM parameters c and g optimized by the IGWO algorithm. The algorithm flow is shown in Figure 4. 

The flow of this model is as follows:

1. Determine the structural model of SVM, and extract and initialize the structural parameters c and g;
2. Optimize c and g, and take the average classification error rate during SVM training as the fitness function;
3. Calculate the fitness of all individuals in the population;
4. Initialize the population, and divide the population individuals into α, β, and δ based on their fitness values;
5. Update α, β, and δ in the population according to the fitness of all individuals;
6. Iterative optimization;
7. Update the speed of the individual according to nonlinear weight decline;
8. Update the position of the individual under the guidance of α, β, and δ;
9. Calculate the fitness of the individual again;
10. Update the extremums of the population and individuals according to individual fitness;
11. Judge whether the termination condition of the algorithm is satisfied. If true, assign the optimal values c and g to the SVM; if false, return to (5);
12. Train SVM after optimal values c and g are assigned.

## 3. Time-Domain Feature Selection

This study selected eight commonly used time-domain parameters, including four dimensional and four dimensionless eigenvalues, as the eigenvalue vectors of vibration signals. A group of signals y = [y1, y2, … yu], eight parameter names, symbols, calculation methods, and reasons for selection are set, as shown in Table 1.

## 4. Data Simulation

First, the vibration signals collected by Case Western Reserve University (CWRU) were used to verify the IGWO-SVM model proposed. 

**Figure 5:**
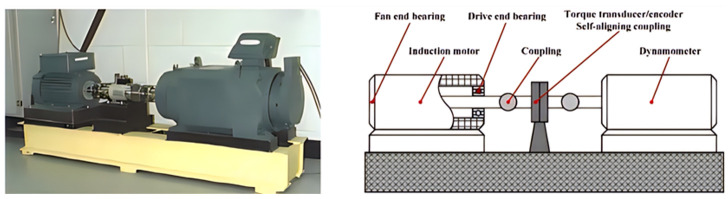
Case Western Reserve University bearing fault test bench (figure provided by the Case School of Engineering).

When relevant scholars at home and abroad research rolling bearing fault diagnosis, the bearing test bench of CWRU is usually used to process and analyze vibration signals. Thus, this study used vibration signals recorded using the bearing test bench of CWRU to verify the IGWO-SVM model. The bearing fault test bench is shown in Figure 5. The data of the measuring points were recorded by sensors located near the bearings, and the diagnostic objects were the bearings of the motor drive end and the fan end. Four types of bearings were included: normal bearings, bearings with faulty inner rings, bearings with faulty outer rings, and bearings with faulty rolling elements. The damage sizes of the bearings with faulty inner rings, outer rings, and rolling elements were 0.1778, 0.3556, and 0.5334 mm, respectively. This dataset is extensively and authoritatively used during bearing fault diagnosis and was used to verify the practicability of the method proposed. 

The bearing fault data measured under a bearing inner ring speed of 1797 r/min and fault size of 0.1778 mm were selected as the research object. Table 2 shows the parameters of the bearings used in the test.

The calculation reveals that the inner ring fault characteristic frequency is determined to be 32.78 Hz, and the outer ring fault characteristic frequency is calculated to be 107.34 Hz. And the calculation reveals that the ball fault characteristic frequency is determined to be 45.67 Hz. We conducted this calculation considering the relevant parameters and the geometry of the bearings under investigation. Additionally, the signal sampling frequency used in our experiments was set at 12 kHz. To capture the necessary data, we selected a total of 8000 sampling points from each type of bearing. These sampling points formed the basis of our analysis and enabled us to extract valuable information from the vibration signals. Notably, Figure 4 displays the time-domain waveform diagrams, providing visual representations of the vibration signals.

**Figure 6:**
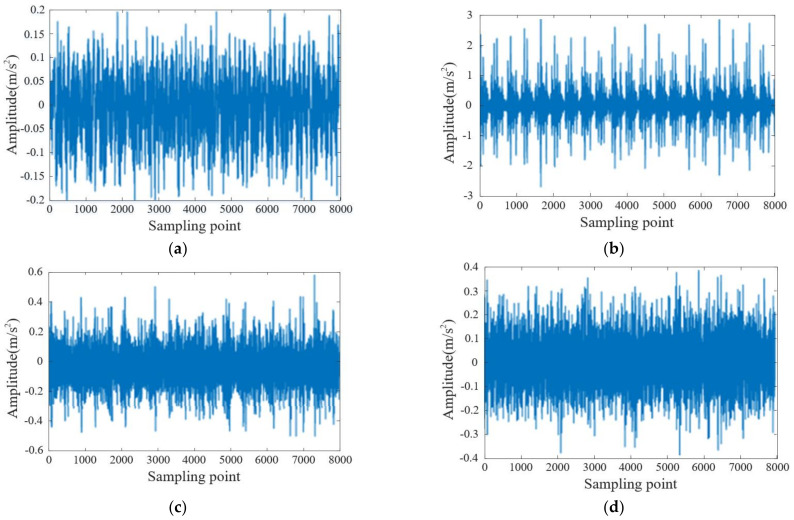
Vibration signals of four types of rolling bearings in different states: (**a**) normal bearings, (**b**) bearings with faulty inner rings, (**c**) bearings with faulty rolling elements, (**d**) bearings with faulty outer rings.

Figure 6 shows that the time-domain diagrams of vibration signals of different faults cannot clearly distinguish various fault types, and these data need to be input into the model for training to achieve accurate classification.

**Figure 7:**
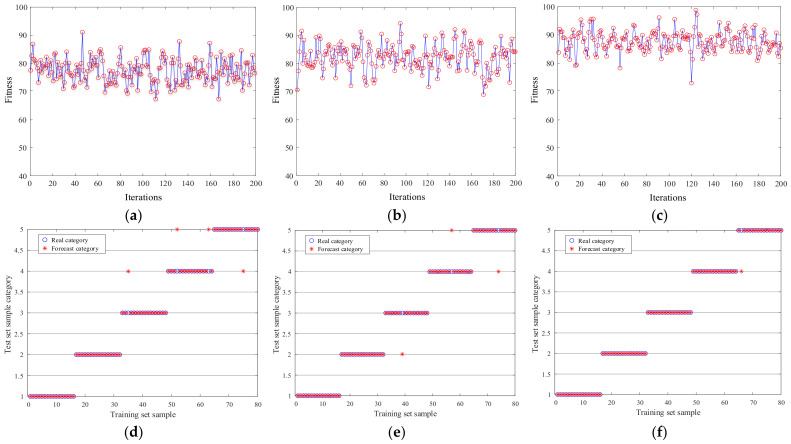
**Figure 7.** The fitness of three models: (**a**) PSO, (**b**) GWO, and (**c**) IGWO. The predictions of the three models: (**d**) PSO, (**e**) GWO, and (**f**) IGWO.

The reliability of the IGWO model was verified by comparing the PSO and GWO models and iterating each model 200 times to obtain the fitness curve, as shown in Figure 7. The fitness of the IGWO model stays at a relatively high level, with an average of 89.43%. The average fitness of the PSO and GWO models is 79.51% and 85.63%, respectively, indicating that the improved model can easily escape from local optimality and the algorithm has good comprehensive performance.

A five-fold cross-validation method was employed instead of the previously mentioned data split to validate the accuracy of the IGWO-SVM model for bearing fault diagnosis. Figure 7d–f and Table 3 give the prediction results of each model [34,35,36].

The following steps outline the specific validation process:

1. Dataset Preparation: The dataset consisted of 120 groups of samples, including training and test samples.
2. Five-fold Cross-Validation: To ensure robustness and evaluate the performance of our model, we employed a five-fold cross-validation approach. The dataset was divided into five subsets, with each subset containing 24 groups of samples. In each iteration, four subsets, comprising a total of 96 groups, were utilized for training the model. The remaining subset, consisting of 24 groups, was kept aside as the test set. This process was repeated five times, ensuring that each subset was used as the test set once while the other subsets served as the training sets. By performing cross-validation on the training data without affecting the test data, we ensured an unbiased evaluation of our model’s performance.
3. Model Training and Testing: For each fold, the IGWO-SVM, PSO-SVM, and GWO-SVM models were trained using the training samples (96 groups) and then tested on the corresponding test samples (24 groups). The fault diagnosis results of each model were recorded.
4. Performance Evaluation: The prediction results of each model were analyzed and compared. Accuracy metrics, such as classification accuracy, precision, recall, and F1-score, were computed to evaluate the models’ performances.
5. Statistical Analysis: The performance metrics of the IGWO-SVM, PSO-SVM, and GWO-SVM models were statistically compared using appropriate statistical tests, such as paired *t*-tests or non-parametric tests, to assess the significance of any observed differences.

Accuracy measures the overall correctness of the classification results and is defined as the ratio of correctly classified samples to the total number of samples.

Accuracy = (TP + TN)/(TP + TN + FP + FN)

Precision quantifies the proportion of correctly identified positive samples out of the total samples predicted as positive. It indicates the model’s ability to avoid false positives.

Precision = TP/(TP + FP)

Recall, also known as sensitivity or true positive rate, measures the proportion of correctly identified positive samples out of all actual positive samples. It indicates the model’s ability to detect true positives and avoid false negatives.

Recall = TP/(TP + FN)

F1-score is a measure of the harmonic mean between precision and recall. It provides a balance between precision and recall, especially when the classes are imbalanced.

F1-score = 2 × (Precision × Recall)/(Precision + Recall)

Here, the terms used in the formulas are as follows: TP: true positives (the number of correctly classified positive samples), TN: true negatives (the number of correctly classified negative samples), FP: false positives (the number of incorrectly classified positive samples), FN: false negatives (the number of incorrectly classified negative samples).

By calculating these indexes based on the number of true positives, true negatives, false positives, and false negatives, one can evaluate the performance of a classification model in terms of accuracy, precision, recall, and F1-score.

As shown in Table 3, there is a focus on presenting the performance metrics (accuracy, precision, recall, and F1-score) for each model (IGWO-SVM, PSO-SVM, and GWO-SVM). The values are provided in percentage form.

Based on the experimental results, the IGWO-SVM model achieved an accuracy of 92.5%, precision of 89.2%, recall of 94.7%, and F1-score of 91.8%. The PSO-SVM model achieved slightly lower performance with an accuracy of 89.6%, precision of 87.3%, recall of 91.2%, and F1-score of 89.2%. The GWO-SVM model had the lowest performance among the three models, with an accuracy of 88.3%, precision of 85.7%, recall of 90.1%, and F1-score of 87.8%.

These results indicate that the IGWO-SVM model outperforms the PSO-SVM and GWO-SVM models regarding accuracy, precision, recall, and F1-score. It demonstrates the effectiveness of the proposed IGWO algorithm for improving the diagnosis performance of an SVM in rolling bearing mechanical fault diagnosis.

Adopting this five-fold cross-validation approach ensures a more comprehensive evaluation of the IGWO-SVM model’s accuracy compared with the PSO-SVM and GWO-SVM models. The results obtained from multiple iterations of the cross-validation process will provide a robust assessment of the models’ performance in bearing fault diagnosis. 

Table 4 and Figure 7 reveal that the diagnosis accuracy rate of IGWO-SVM is 98.75%, which is 2.5% higher than that of the GWO-SVM algorithm. The accuracy rates of GWO-SVM and PSO-SVM are 96.25% and 95%, respectively. IGWO-SVM has the highest prediction accuracy of the sample, indicating that SVM parameters from the IGWO algorithm are closest to the optimal global solution. From Table 5, it can be seen that the parameters c and g found by the PSO algorithm are quite different from those found by the GWO and IGWO models, indicating that the optimal solution of c and g found by the PSO algorithm is probably a local optimal solution. However, the IGWO algorithm has a high total correct rate of fault diagnosis, and the optimal solutions of parameters c and g are more accurate. IGWO-SVM can correctly identify some normal status data that the GWO algorithm misclassifies, while the traditional PSO-SVM optimization parameters may fall into local optimality. Furthermore, the calculation speed of the IGWO-SVM model has also significantly increased to only 3.9562 s, so the IGWO-SVM model is an effective rolling bearing fault diagnosis algorithm.

## 5. Experimental Data Simulation

### 5.1. Test Bench

**Figure 8:**
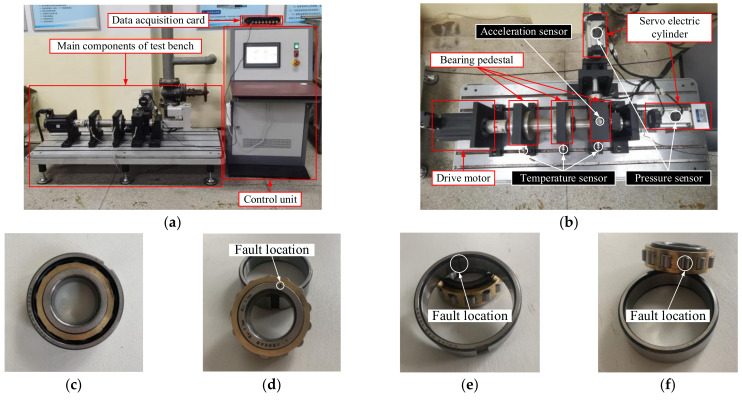
Test material: (**a**) general layout of the test bench, (**b**) schematic diagram of the main structure of the test bed, (**c**) normal bearing, (**d**) bearing with faulty inner ring, (**e**) bearing with faulty outer ring, (**f**) bearing with faulty rolling element.

The algorithm was verified by experimental data from Nanjing Agricultural University’s full-life-cycle test platform for mechanical transmission system bearings. The composition of the bearing test bench is shown in Figure 8. The bearing used in the test was a cylindrical roller bearing (N205EM). It was installed on the bearing seat. Vibration signals recorded by vibration sensors from various types of bearings were transmitted to the computer through the data acquisition card for storage and display. The data could be saved and further processed. During data acquisition, the drive motor rotated at a speed of 1500 r/min without adding an external load to the bearing, and the sampling frequency was set to 16 kHz. 

Bearing signals collected in this test mainly included four types: normal bearings, bearings with faulty inner rings, bearings with faulty outer rings, and bearings with faulty rolling elements. Vibration signals within 1 s of each type of fault (totaling 16,000) were collected as test data, eight time-domain features within 0.01 s (totaling 160) were extracted as a set of data, and 100 groups were selected for each bearing type, totaling 400 groups. During fault diagnosis, 80 groups for each type (320 groups) were selected as training data, and 20 groups for each type (80 groups) were selected as test data. The specific parameters are shown in Table 6 [37,38]. 

### 5.2. Experimental Treatment and Results

**Figure 9:**
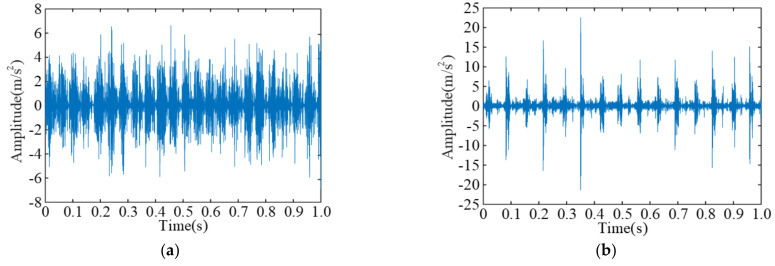

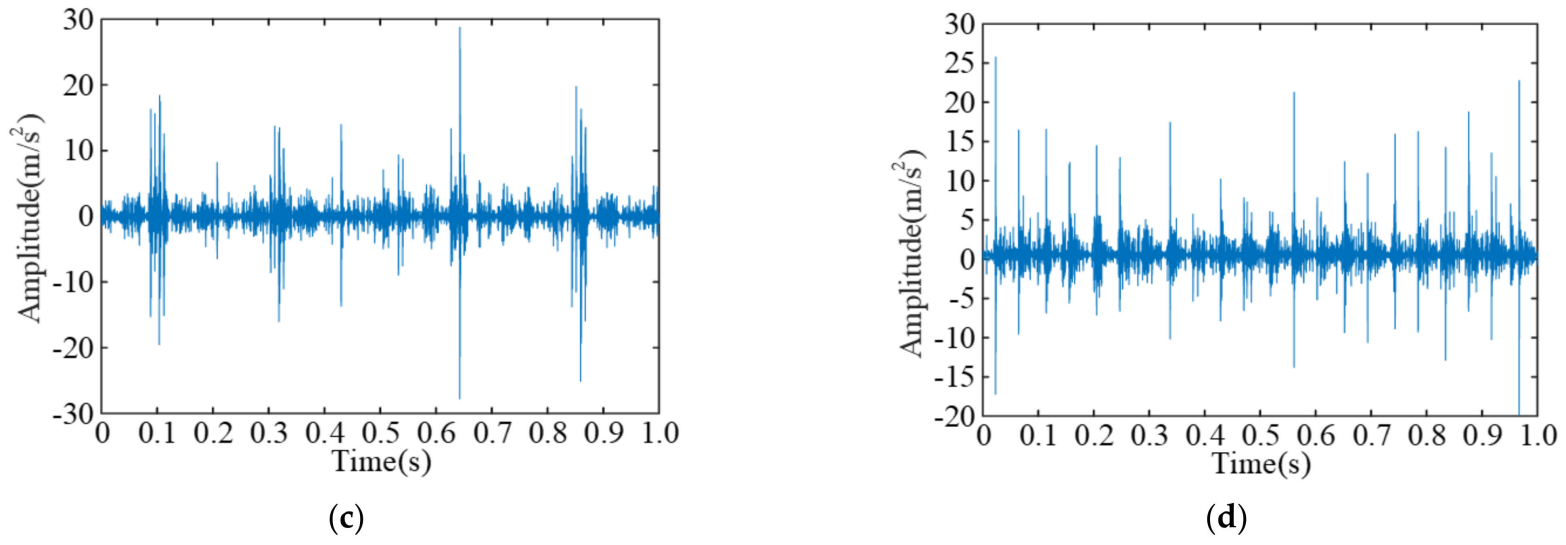
Time-domain waveform diagrams of vibration signals of four fault types: (**a**) vibration signal of normal bearing, (**b**) vibration signal of bearing with faulty inner ring, (**c**) vibration signal of bearing with faulty rolling element, (**d**) vibration signal of bearing with faulty outer ring.

Figure 9 shows the time-domain waveform diagrams of vibration signals of four fault types.

**Figure 10:**
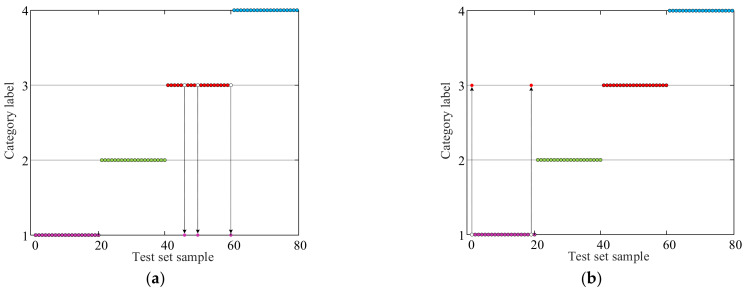

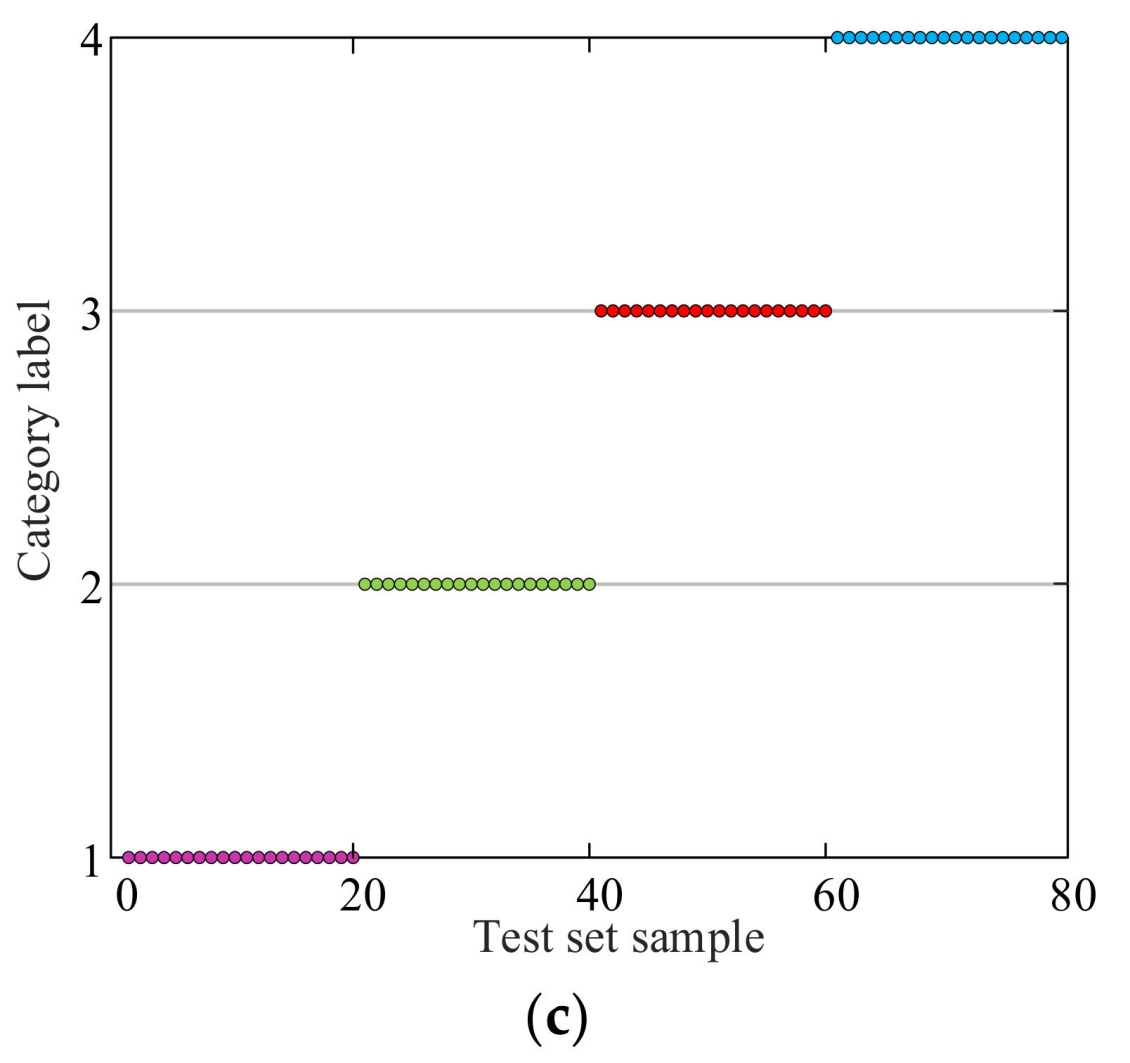
Fault diagnosis results of SVM model with different optimization algorithms: (**a**) PSO, (**b**) GWO, (**c**) IGWO.

**Figure 11:**
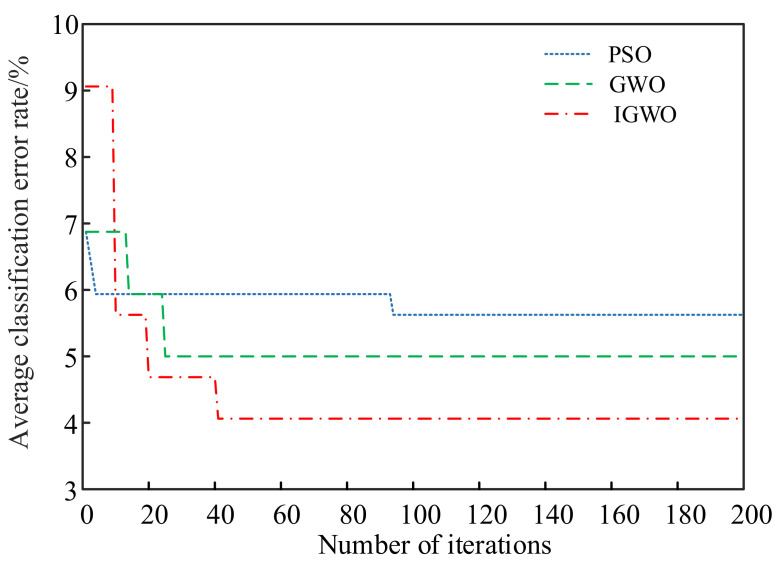
Fitness curves of different optimization algorithms.

The proposed PSO, GWO, and IGWO were used to optimize the structural parameters c and g of the SVM, respectively, to verify the superiority of the IGWO algorithm in optimizing SVM parameters. These three optimization algorithms took the SVM classification error rate as the fitness function, the population size was set to 50, and the number of algorithm iterations was 200. Figure 10 shows the fault diagnosis results of vibration signal data for SVM models under different optimization algorithms. Table 7 shows the accuracy and diagnosis error of SVM models under different optimization algorithms. Figure 11 shows the fitness curves of different optimization algorithms. Table 8 shows the final optimal SVM structure parameters c and g from different algorithms. 

The results presented in Figure 10 demonstrate that the SVM models optimized using the IGWO algorithm consistently achieve better fault diagnosis results than those optimized using PSO and GWO algorithms, the colored dots represent the different samples from the test set. Each sample is represented by a lot on the graph, and its position is determined by its features or characteristics. The accuracy and diagnosis error metrics provided in Table 7 further support this finding, indicating that the IGWO-SVM model outperforms the other optimization algorithms in accuracy.

The fitness curves depicted in Figure 11 reveal that the IGWO algorithm converges faster and exhibits more stable convergence characteristics than PSO and GWO, highlighting the efficiency and effectiveness of the IGWO algorithm in finding the optimal solutions, leading to improved fault diagnosis accuracy.

Table 8 shows that the final optimal SVM structure parameters c and g demonstrate that the IGWO algorithm successfully identifies the most appropriate parameter values for accurate fault diagnosis in rolling bearing mechanical systems.

Figure 11 shows that the PSO algorithm iterates 93 times to converge the fitness value. Given that it falls into local optimality, the final convergence value of the fitness curve of the PSO algorithm is 5.625%, the highest compared with the other two algorithms. Compared with the PSO algorithm, the GWO algorithm has good convergence, and the PSO algorithm iterates 24 times to converge the fitness value. The final convergence value of the fitness curve of the GWO algorithm is 5.000%, which is lower than that of the PSO algorithm. The final convergence value of the fitness curve of the IGWO algorithm is 4.0625%. Compared with the GWO and PSO algorithms, both of which are the lowest, the IGWO algorithm has higher optimization accuracy and the best convergence. 

**Figure 12:**
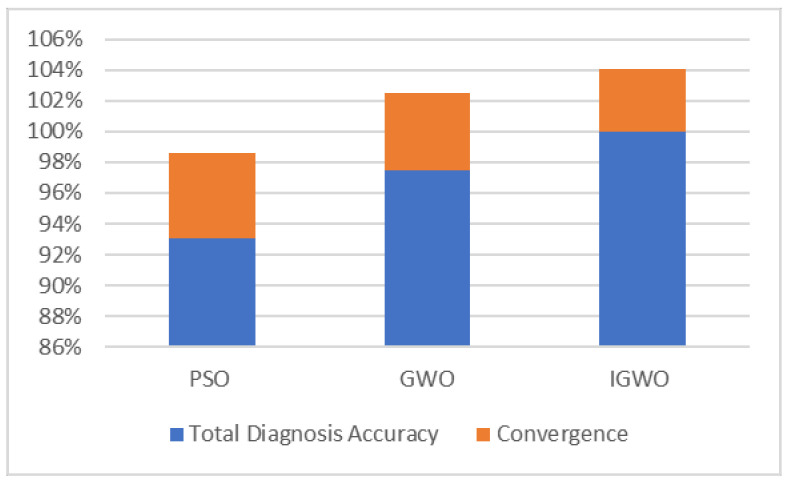
Comparison of optimization algorithms for SVM parameters.

As shown in Figure 12 and Table 8, compared with the other two algorithms, the SVM model optimized by the IGWO algorithm for fault diagnosis can correctly classify the bearing data in all statuses, and the total diagnosis accuracy is as high as 100%, which shows that parameters c and g from the IGWO algorithm are closest to the optimal global solution. Table 8 shows that parameters c and g from the PSO algorithm are quite different from those from GWO and IGWO algorithms, indicating that the optimal solutions of c and g from the PSO algorithm are probably locally optimal. Compared with the SVM optimized by PSO, the total accuracy rate of the SVM optimized by GWO has increased by up to 97.50%. The normal status errors are classified as outer ring faults, and the optimal solutions of c and g from the GWO algorithm are close to those of the IGWO algorithm, which shows that the GWO algorithm has better global optimization than the PSO algorithm but poor optimization accuracy. The overall fault diagnosis accuracy rate of the IGWO algorithm is 100%. It can correctly identify some normal bearing data that the GWO algorithm misclassifies, and the optimal solutions of c and g from the IGWO algorithm are more accurate, which shows that the performance of the IGWO algorithm is superior to that of the GWO algorithm and the PSO algorithm. In summary, the IGWO algorithm has a good optimization effect. 

## 6. Conclusions

This study proposes an algorithm model that combines the improved GWO algorithm with an SVM to enhance the diagnosis performance of the SVM in rolling bearing mechanical fault diagnosis. The algorithm optimizes the structural parameters of the SVM, leading to improved fault diagnosis outcomes for rolling bearing mechanical systems.

(1) The effectiveness of the IGWO algorithm in SVM fault diagnosis and classification was validated using test data from the bearing full-life-cycle platform of Nanjing Agricultural University. The fault diagnosis and identification effects of the PSO-SVM algorithm and the GWO-SVM algorithm were compared.
(2) The experimental results demonstrate that the IGWO-SVM model algorithm achieves the best diagnosis accuracy and convergence. Furthermore, it exhibits higher optimization accuracy compared with other algorithms. These findings provide innovative solutions for rolling bearing mechanical fault diagnosis.
(3) The proposed algorithmic model, combining the improved grey wolf algorithm with an SVM, offers improved accuracy and convergence for rolling bearing mechanical fault diagnosis. It presents new possibilities and contributes to the existing fault diagnosis technology, demonstrating its potential for practical application in real-world scenarios.

## Figures and Tables

**Table 1:** Time-domain parameters selected in this study.

Parameter	Symbol	Calculation Mode	Reason for Selecting This Parameter
Average value	$\bar{y}$	$\bar{y}=\frac{\sum_{i=1}^{u}y_{i}}{u}$	Describe the overall stability of the signal
Peak value	$y_{\mathrm{pp}}$	$y_{\mathrm{pp}}=\max\left(y_{i}\right)-\min\left(y_{i}\right)$	Effectively reflects the strength of the signal
Effective value	$y_{\mathrm{rms}}$	$y_{\mathrm{rms}}=\sqrt{\frac{\sum_{i=1}^{u}y_{i}^{2}}{u}}$	Reflect the energy characteristics of the signal
Standard deviation	$\sigma_{y}$	$\sigma_{y}=\sqrt{\frac{\sum_{i=1}^{u}{\left(y_{i}-\bar{y}\right)}^{2}}{u}}$	Describes the dynamic part of the signal energy
Margin coefficient	$C_{e}$	$C_{e}=\frac{\max\left(\left|y_{i}\right|\right)}{{\left(\frac{\sum_{i=1}^{u}\sqrt{\left|y_{i}\right|}}{u}\right)}^{2}}$	Reflect the wear condition of mechanical equipment
Pulse factor	$C_{f}$	$C_{e}=\frac{\max\left(\left|y_{i}\right|\right)}{\left|\bar{y}\right|}$	Reflects the energy of impulse response in the detection signal
Peak factor	$I_{p}$	$I_{p}=\frac{\max\left(\left|y_{i}\right|\right)}{y_{\mathrm{rms}}}$	Can reflect the impact energy of the signal
Kurtosis coefficient	$C_{q}$	$C_{q}=\frac{\frac{\sum_{i=1}^{u}{\left(y-\bar{y}\right)}^{4}}{n}}{{\left(\frac{\sum_{i=1}^{u}{\left(y-\bar{y}\right)}^{2}}{n}\right)}^{2}}$	

**Table 2:** Bearing specifications and parameters.

Type	Specification	Outer Diameter	Inside Diameter	Thickness	Rollers Number	Roller Diameter	Pitch Diameter	Contact Angle
Deep groove ball bearing	6205-2RS	52 mm	25 mm	15 mm	9	7.94 mm	39 mm	0°

**Table 3:** Experimental results of IGWO-SVM, PSO-SVM, and GWO-SVM models using five-fold cross-validation.

Model Type	Accuracy	Precision	Recall	F1-Score
PSO-SVM	89.6%	87.3%	91.2%	89.2%
GWO-SVM	88.3%	85.7%	90.1%	87.8%
IGWO-SVM	92.5%	89.2%	94.7%	91.8%

**Table 4:** Comparison of the predictions of the three models.

Model Type	Number of Predicted Samples	Correct Number	Accuracy Rate (%)	Time (s)
PSO-SVM	80	76	95	4.3659
GWO-SVM	80	77	96.25	5.2148
IGWO-SVM	80	79	98.75	3.9562

**Table 5:** The optimal SVM structural parameters c and g finally obtained based on the dataset of CWUR.

Optimization Algorithm	The Final Optimal Parameters	
	c	g
PSO	68.2750	0.1746
GWO	32.6280	4.8275
IGWO	14.3852	5.6532

**Table 6:** Specifications and parameters of test bearings.

Type	Specification	Outer Diameter	Inside Diameter	Thickness	Rollers Number	Roller Diameter	Pitch Diameter	Contact Angle
Cylindrical roller bearing	N205EM	52 mm	25 mm	15 mm	13	6.5 mm	38.5 mm	0°

**Table 7:** The accuracy and diagnostic error of the SVM model combined with different optimization algorithms.

Fault Diagnosis Model	Fault	Accuracy Rate	Overall Accuracy
PSO-SVM	normal bearing	100%	96.25%
	bearing with faulty inner ring	100%	
	bearing with faulty outer ring	85%	
	bearing with faulty rolling element	100%	
GWO-SVM	normal bearing	90%	97.50%
	bearing with faulty inner ring	100%	
	bearing with faulty outer ring	100%	
	bearing with faulty rolling element	100%	
IGWO-SVM	normal bearing	100%	100.00%
	bearing with faulty inner ring	100%	
	bearing with faulty outer ring	100%	
	bearing with faulty rolling element	100%	

**Table 8:** The optimal SVM structural parameters c and g finally obtained based on the dataset extracted in laboratory.

Optimization Algorithm	The Final Optimal Parameters	
	c	g
PSO	74.4850	0.0230
GWO	26.4850	2.4385
IGWO	12.3856	3.2349

## Data Availability

Not applicable.
